# Graft-Transmitted siRNA Signal from the Root Induces Visual Manifestation of Endogenous Post-Transcriptional Gene Silencing in the Scion

**DOI:** 10.1371/journal.pone.0016895

**Published:** 2011-02-09

**Authors:** Atsushi Kasai, Songling Bai, Tianzhong Li, Takeo Harada

**Affiliations:** 1 Faculty of Agriculture and Life Science, Hirosaki University, Hirosaki, Japan; 2 Laboratory of Fruit Cell and Molecular Breeding, China Agriculture University, Beijing, China; Instituto de Biología Molecular y Celular de Plantas, Spain

## Abstract

In plants, post-transcriptional gene silencing (PTGS) spreads systemically, being transmitted from the silenced stock to the scion expressing the corresponding transgene. It has been reported that a graft-transmitted siRNA signal can also induce PTGS of an endogenous gene, but this was done by top-grafting using silenced stock. In the present study involving grafting of *Nicotiana benthamiana*, we found that PTGS of an endogenous gene, *glutamate-1-semialdehyde aminotransferase* (*GSA*), which acts as a visible marker of RNAi via inhibition of chlorophyll synthesis, was manifested along the veins of newly developed leaves in the wild-type scion by the siRNA signal synthesized only in companion cells of the rootstock.

## Introduction

Much like the blood vessels of animals, the vascular system of plants provides a pathway by which important nutrients and water can move from one part of the plant body to another. Furthermore, both the subterranean root and the aerial parts of plants must communicate to achieve accommodative growth. This task was solved by the evolution of a system for long-distance transport of signals, such as plant hormones, through the sieve tubes [1]–[3]. Recent advances in analytical technology have revealed that an abundance of mRNAs and small RNAs exist in the phloem sap [4], [5] and some RNAs are transported over long distances through the sieve tube to function at sites where they are required [4], [5], and some of the data have been obtained through grafting experiments [6]. Grafting is a technique for fusing materials from two individual plants possessing different genomes [7]. The bottom part of the plant, which contributes roots and support, is called the rootstock, and the upper part, contributing stems, leaves, flowers, and fruits, is called the scion. This technology is widely employed in horticultural crops for vegetative propagation, avoidance of juvenility, size control, and acquisition of biotic stress resistance [7]. When a graft takes successfully, the two separate plants having different genomes appear to grow as a single unit. Therefore, in grafted plants, a vascular connection between the scion and the rootstock is an essential prerequisite.

The mobile siRNA signal through sieve tube in plants functions as an effective defense mechanism against viral RNA molecules [8], because the signal movement either with or ahead of the virus ensures that the virus cannot escape the effects of silencing in the whole plant body. As a matter of course, it is anticipated that an artificial siRNA signal from rootstock plant given the potency for the siRNA production can function for the PTGS of gene possessing the same sequence with the siRNA in the scion. Recently, an exhaustive analysis using *Arabidopsis* and deep sequencing has revealed that siRNA derived from endogenous inverted repeat loci can pass through the graft union and serve a function in recipient cells [9], [10]. Therefore, practical research on the combination of siRNA transport system and grafting technology is of current interest [6].

Palauqui and Vaucheret [11] reported that the transcript of endogenous nitrate reductase gene over-accumulate above the level of wild-type was undergone the PTGS by grafting onto the silenced stock. Voinnet et al. [12] also reported that a virus-induced gene silenced (VIGS) signal stock can induce silencing of two endogenous genes, *phytoene desaturase* and *ribulose bisphosphate carboxylase small subunit*, in the scion without the virus replication there. VIGS, however, is considered to be undesirable for practical system, because there is a risk for the potential of generating new infectious viruses by recombination and mutation which are generated by errors during the replication of genomes [13]. Furthermore, both reports [11], [12] were performed by top grafting, using silenced stock plants having much branches. We report here that companion cell specific production of siRNA signal in only rootstock can introduce a visual manifestation of an endogenous PTGS in the grafted partner, scion.

## Results and Discussion

### siRNA-overexpressing transgenic plants

**Figure 1:**
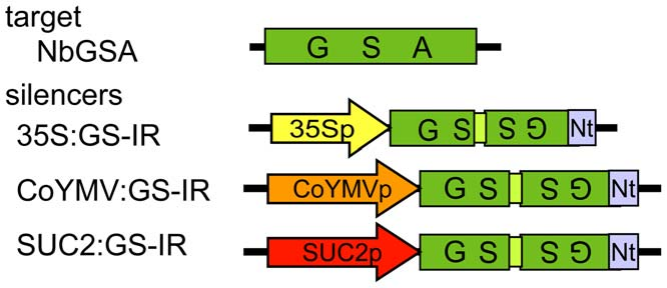
Schematic diagrams of the constructs used to produce the siRNA of *GSA* gene. *N. benthamiana GSA* cDNA as the endogenous target gene is also shown. The cauliflower mosaic virus 35S promoter (35Sp), Commelina Yellow Mottle Virus promoter (CoYMV), or Sucrose transporter AtSUC2 gene promoter (SUC2p) directed the expression of GS-IR (the part of GSA inverted repeat) transgene.

To allow visual detection of endogenous PTGS, we made a *glutamate-1-semialdehyde aminotransferase* (*GSA*) gene silencing-inducing construct from *NtGSA* [14], a useful visible marker in *N. tabacum* via inhibition of chlorophyll synthesis, which shows high nucleotide sequence homology to the ortholog *GSA* gene of *N. benthamiana* (Figure S1). The inverted 689-bp repeat structure of *NtGSA2* cDNA (139-828 of accession No. x65974) was linked to the cauliflower mosaic virus (CaMV) 35S promoter, phloem companion cell-specific commelina yellow mottle virus (CoYMV) promoter [15], and the Arabidopsis sucrose transporter (AtSUC2) promoter [16] named 35S:GS-IR, CoYMV:GS-IR, and SUC2:GS-IR, respectively (Figure 1). The transgenic *N. benthamiana* obtained using 35S:GS-IR exhibited variegated pale-green leaves as in the case of *N. tabacum* [14], and the CoYMV:GS-IR transgenic leaves had pale-green leaf veins (Figure S2). In the latter, the cells surrounding the leaf veins contained a lower amount of chlorophyll pigment than those of the control. Furthermore, quantitative RT-PCR analysis revealed that the levels of *NbGSA* transcripts were inversely proportional to the siRNA levels in the leaves, while mRNA level of *NbSU-s* [17] as the control gene was not altered in these plants (Figure S2), indicating that PTGS of *GSA* expression was clearly dependent upon the level of *GSA* siRNA.

### Analyses using agroinfiltration

**Figure 2:**
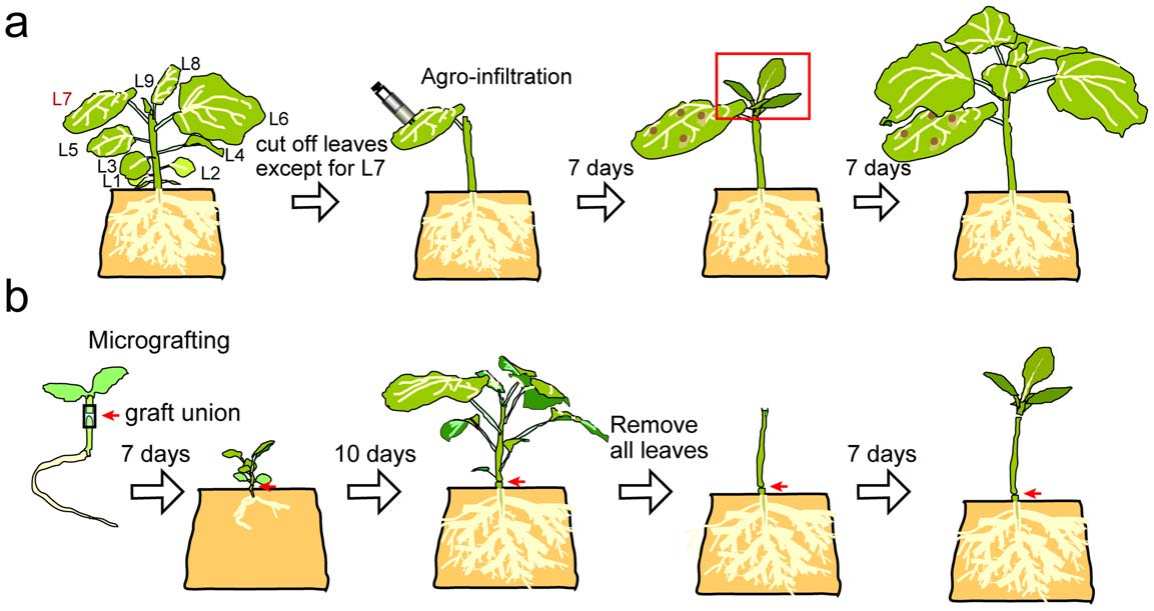
Illustration of PTGS experiments using agroinfiltration and micrografting. (a) A four-week-old plant was stripped of all the lateral leaves except for L7 to encourage the establishment of a source L7 leaf and a sink for newly developing leaves. Seven days later, the leaves that emerged (marked by square) from the apex were sampled. (b) Five-day-old seedlings were grafted using a silicone tube. After 7 days of culture on MS agar, the tube was removed, and then the graft plant was transferred to rockwool soaked with nutrient solution. After 10 days, all the leaves were cut off to encourage the establishment of a root source and emerging leaf sink, and then after another 7 days the newly developed leaves were observed and sampled.

**Figure 3:**
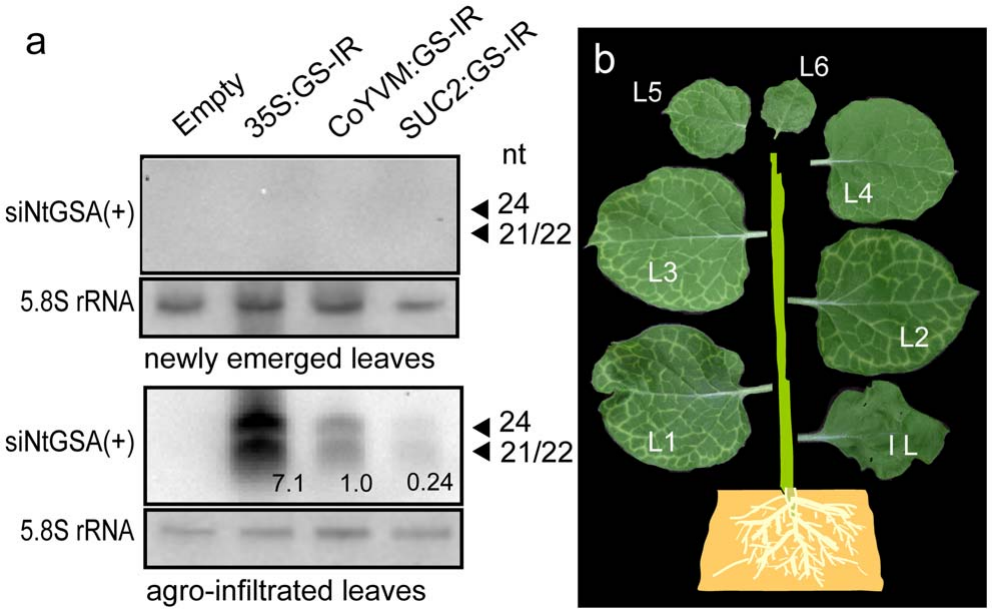
RNA gel blot analysis of *GSA* siRNA in agroinfiltrated WT and manifestation of PTGS in newly emerged leaves. (a) IL after 7 days of agroinfiltration and newly emerged L1 were used as agroinfiltrated leaves and newly emerged leaves, respectively. The numbers in the result of agroinfiltrated leaves indicate the relative levels of 24-nt siRNA calculated the respective rRNA signal intensities. (b) Leaves developed newly from shoot apex at the 14 days post infiltration are arranged digitally for an easy comprehensible manner.

To clarify the effect of the *GSA* siRNA signal transported over a long distance, an agroinfiltration experiment combined with a procedure (Figure 2a) to enhance source and sink power [18], [19] was performed. Although the levels of *GSA* siRNA in the infiltrated leaves were most abundant for 35S:GS-IR, followed by CoYMV:GS-IR and SUC2:GS-IR (Figure 3a), clear pale-green cells along the veins of newly emerged leaves were apparent only in CoYMV:GS-IR (Table 1). Unequivocal PTGS in CoYMV:GS-IR was also confirmed by the reduction in the amount of chlorophyll (Figure S3). The manifestation intensity gradually decreased in the leaves with subsequent development (Figure 3b). Although a considerable difference (approximately 30-fold) in the levels of siRNA was observed between the 35S:GS-IR- and SUC2:GS-IR-infiltrated leaves, both exhibited slightly pale-green veins in the proximal portions in about half of the plants tested (Table 1), indicating that companion cell-specific expression of GS-IR under the SUC2 promoter was effective for distant induction of PTGS, and that the CoYMV promoter was able to provide the siRNA more effectively than SUC2. Overall, these results demonstrated that the siRNA signal was transported over a long distance from the companion cells of the infiltrated leaf, and then unloaded from the sieve tube of the newly developed leaf, resulting in PTGS in the cells located in the vicinity of the leaf vein.

**Table 1:** Manifestation of GSA gene silencing in the new first leaf emerged.

Construct usedor transgenic plant as stock	Numbers oftested plants	Numbers ofsilencing manifested plants
AgroinfiltrationEmpty35S:GS-IRCoYMV:GS-IRSuc2:GS-IR	11111111	05*115*
GraftingEmpty35S:GS-IRCoYMV:GS-IR	867282	0026

*manifested at only distal portion of the leaf.

### Manifestation of PTGS by grafting

**Figure 4:**
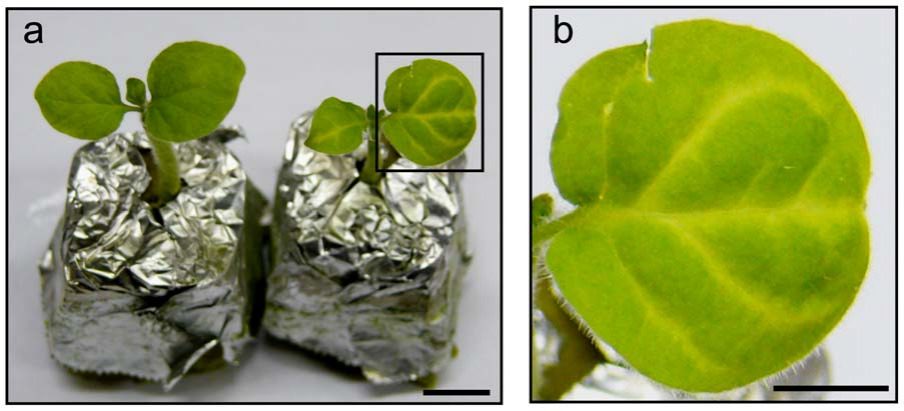
Manifestation of *GSA* PTGS in newly developed leaves of grafted plant. (a) Newly emerged leaves of grafted plants. WT on Empty rootstock (left), WT on CoYMV:GS-IR rootstock (right). Bar indicates 1 cm. (b) Enlarged view of the inlay in (a). Bar indicates 0.5 cm.

We then performed grafting experiments using the transgenic 35S:GS-IR or CoYMV:GS-IR *N. benthamiana* as stock and the wild type as the scion (Figure 2b). The respective lines exhibiting the most distinctive PTGS and harboring only one transgene copy were selected from among several transgenic plants. Approximately 30% of newly developed leaves of the wild type (WT) scion on CoYMV:GS-IR showed *GSA* PTGS, as in the case of agroinfiltration, whereas no silencing was observed in the case of the WT on 35S:GS-IR (Figure 4, Table 1), again indicating that the CoYMV promoter was effective for facilitating long-distance transport of the siRNA signal. No manifestation of *GSA* PTGS was evident on leaves that developed after the 3rd leaf (data not shown), indicating earlier disappearance of the manifestation than was the case for agroinfiltration. This difference was probably due to the fact that strong transient expression of GS-IR by agroinfiltration was able to provide a much more effective siRNA signal for PTGS than that from the transgenic rootstock.

### Absence of siRNA amplification system

Brosnan et al. [20] showed that graft-transmissible mRNA silencing is executed primarily on sequences downstream of those homologous to the mobile signal using a transgene. To clarify whether this siRNA amplification system would facilitate endogenous *GSA* PTGS, we analyzed the transitivity of RNA silencing [21] along the target sequence. Northern blotting using a probe for the 3′ flanking region of the *GSA* target showed no signal in either the 35S:GS-IR or the CoYMV:GS-IR transgenic line (Figure S4), although both exhibited unequivocal PTGS (Figure S2a, S2b). As the same results were obtained even from agroinfiltrated leaves, it is likely that the *GSA* siRNA molecules derived from the IR- sequence of our plasmids was transported into the newly developed leaves, and could not have been amplified through the action of RNA-dependent RNA polymerase, which would account for whole-plant-body systemic PTGS. As a result, even the siRNA molecules corresponding to the GS-IR sequence could not be detected in the newly emerged leaves by northern hybridization (Figure 3a, S4b).

### Future perspective

Our present data clearly show that a siRNA signal transported over a long distance can induce endogenous PTGS. It seems likely that strong expression in companion cells by the CoYMV promoter [15] and enhancement of the sink power contributed to these achievements. It has been reported that movement of the silencing signal is more efficient from shoot to root than *vice versa*, consistent with the source to sink movement of viruses and assimilates [10]. As the PTGS of the target gene *GSA* was manifested exclusively in leaves, we did not analyze the movement of the siRNA signal from shoot to root. More effective endogenous PTGS would occur if a gene, whose silencing leads a phenotypic change in the root, were targeted by siRNA from the shoot. We are currently studying target genes that are expressed actively in the root system, especially in phloem, such as *GIBBERELLIC ACID-INSENSITIVE* [22] and *SOLITARY-ROOT/IAA14* [23].

Non-cell-autonomous miRNA regulates a wide variety of developmental processes [24], [25] in plants. Furthermore, some miRNAs function as physiological signals that are transported over long distances between organs, such as leaves and roots [26]–[29]. On the basis of the present findings, we propose that a system for long-distance transport of artificial siRNA like the one used here would be useful for regulating the expression of specific genes in crops. This approach would seem especially attractive for fruit trees such as apple, because they are widely cultivated using grafting over periods of several decades [6]. Transgenic stock that is competent for transport of a specific siRNA would provide an innovative technique for improving the agricultural characteristics of a grafted scion cultivar.

## Materials and Methods

### Plant materials and growth conditions

Transgenic *N. benthamiana* was obtained by *Agrobacterium*-mediated transformation. These transgenic lines were identified by a 3∶1 segregation for kanamycin resistance in T1 seeds. The plants were grown at 24°C under a 16-light/8-h dark cycle with cool fluorescent light at about 100 µmol m^−2^S^−1^.

### Construction of binary vectors

The part of *NtGSA* cDNA (139 to 994 of accession no. X65974), amplified by PCR using primers GSAa and GSAb (Table S1), was subcloned into the *Bam*HI and *Xba*I sites of pBluescript II SK+ plasmid (Stratagene). To this plasmid, the additional *NtGSA* cDNA (139 to 939) amplified by PCR using primers GSAc and GSAd (Table S1) was integrated into the *Xba*I/*Sac*I sites, resulted in the forming of an inverted repeat of the GSA fragment (GS-IR). The GS-IR was inserted *Bam*HI/*Sac*I sites of the binary plasmid pE2113-GUS [30] to replace the *beta-glucuronidase* (*GUS*) gene. The resulting plasmid was used as the 35S:GS-IR (Figure 1). Then the companion-cell-specific promoter, CoYMVp, of pCOI [15], from Prof. Neil Olszewski, University of Minnesota, St. Paul, MN, USA), was amplified by PCR using primers PCoYMVF and PCoYMVR (Table S1), then replaced the promoter of the 35S:GS-IR using the *Sal*I/*Bam*HI sites. The resulting plasmid was used as the CoYMV:GS-IR (Figure 1). Another companion-cell specific promoter AtSUC2 (accession no. X79702), amplified by PCR using primers PSUC2F and PSUC2R (Table S1), was also integrated into the *Sal*I/*Bam*HI sites, resulted in the SUC2:GS-IR (Figure 1). Construction of Empty vector was previously described [31]. DNA sequences of each plasmid were confirmed.

### Agroinfiltration experiments

*Agrobacterium tumefaciens* strain EHA105 carrying one of the constructs (Figure 1) was grown overnight at 28°C in Luria-Bertani(LB) medium with the appropriate antibiotics and 10 µM acetosyringone. The bacteria were briefly spun down (5,000 g, 15 min, RT) and resuspended in suspension buffer (10 mM MES-KOH, pH 5.2, 10 mM MgCl_2_, 100 µM acetosyringone) to an OD600 of 0.1 and left for at least 3 h at room temperature. One month-old *N. benthamiana* plants were cut off the 1st to the 9th leaves with their lateral buds except for the 7th, resulting leaving only 7th leaf and apical bud. Twenty µl of the *agrobacterium* suspension was infiltrated into four sites, at equal spaces each other, of the 7th leaf via a needle-less 1-ml syringe. After agroinfiltration, the plants were kept in the growth room for 7 days. Newly emerged and infiltrated leaves were sampled for the northern blot analysis (Figure 3a). The plants kept for another 7 days were photographed (Figure 3b).

### Micrografting experiments

As shown in Figure 2b, the 7-day-old seedling of *N. benthamiana* germinate on MS [32] agar (0.7%) for 1 week were used for hypocotyl-hypocotyl micrografting. The rootstock donor hypocotyl at approximately 5 mm below the cotyledon was cut horizontally and the cut side of the root part was inserted into a silicone tube (2 mm length, 0.5 mm external×0.4 mm internal diameter, TechJam, Osaka, Japan). The cut surface of scion partner prepared by the same way was adhered against that of the root part of the midway in the tube. All grafting procedures were performed under a stereomicroscope on a clean bench. The grafts were grown on MS agar in Petridishes by setting up by an agarose block (3 mm cube). After 14 days, the tube was cut off from the graft interface and then the grafted plants was transferred to a rockwool (Nitto Bosek Co, Tokyo, Japan) in a standard nutrient solution (Otsuka House No. s.1 and 2, Otsuka Chemical Co., Osaka, Japan). The plants grown for 10 days were cut off all lateral leaves except for the L7 and the shoot apex, and then these were covered the whole with a plastic film (Saran wrap, Asahi Kasei Chemical Co., Tokyo, Japan) to avoid drying, and grown for 7 days, gradually opening it.

### Total RNA extraction and qRT-PCR analysis

Total RNA was extracted from leaves using the phenol/chloroform method essentially as described previously [16]. Residual genomic DNA was eliminated with a TURBO DNA-free Kit (Ambion Inc., Austin, Texas, USA). The cDNAs used for qRT-PCR of *NbGSA* [18] and *NbSu-s* (accession no. AJ571699) were synthesized from 1 µg of total RNA with a SuperScript VILO cDNA Synthesis Kit (Bio-Rad Laboratories, Inc. Hercules, CA, USA) with a Chromo4 real-time PCR detector (Bio-Rad). Fold change of *NbGSA* and *NbSu-s* in the transgenic plants was determined in triplicate and normalized using *NbUbi* (accession no. AY912494) as a standard. The primers used for qRT-PCR are described in Table S1. PCR product melting curves confirmed specificity of single-target amplification, and PCR products were sequenced.

### Extraction of small RNAs and detection of siRNAs

Extraction of small RNAs was performed essentially as described previously [16]. Fifteen micrograms of small RNA-enriched nucleic acids were separated on 15% acrylamide gels with 8 M urea and then transferred to the membrane (Biodyne PLUS, PALL Corporation). The positions for 20- and 30- nucleotide RNA oligomer (DynaMaker Small RNA II, BioDynamics Laboratory) were determined by staining the gels with ethidium bromide. The digoxigenin-labeled *NtGSA* sense and antisense riboprobes were synthesized using DIG RNA labeling Mix and T3, T7 RNA polymerase (Roche Diagnostics GmbH Mannheim, Germany), respectively. The riboprobes were hybridized to small RNAs at 45°C in the DIG Easy Hyb solution (Roche). The membrane was washed twice with 2 X SSC at room temperature and then twice with 2 X SSC/0.5% SDS at 55°C. Hybridized probe was visualized and quantified using a Quantity One (Bio-Rad). The relative amount of siRNAs was calculated by dividing the siNtGSA (24 nt) band counts by the 5.8S rRNA (accession no. AJ492409) counts on the same filter.

### Microscopic observation

Tissue samples were collected along the minor veins near leaf apex, then fixed for 30 minutes at room temperature in a 0.1 M phosphate buffer (pH 7.0) containing 1% glutaraldehyde, and embedded in 7% low melting-point agarose and sectioned (100 µm thick) transversally with a razor blade using a vibratome (Series 1500 Leica St. Louis, MO). Each sample was monitored with a biological fluorescent microscope (BX61, Olympus, Tokyo, Japan), and the digital images of them were captured with a digital camera (DP71, Olympus) connected to the microscope. For the chlorophyll fluorescence imaging, confocal laser scanning microscopy system FluoVie 1000 (Olympus, Tokyo, Japan) was used. A 473-nm diode laser and a 655–755 nm band-pass filter were used for excitation and detection of chlorophyll fluorescence.

### Chlorophyll determination

Chlorophyll was measured in dimethylformamide extracts and concentrations were determined as described previously [33]. Fourteen days after agroinfiltration, three leaf discs (1.0 cm) per a leaf were taken for chlorophyll determination.

## Supporting Information

Figure S1 **cDNA alignment between** ***N. tabacum GSA2*** **(Accession No. x65974) and** ***N. benthamiana*** **orthologous GSA.** Amplified region of *NtGSA* by primers a and b was used as the inverted repeat of the GSA; GS-IR. The sequence from 701 to 751 was the loop frame. On mismatched base pairs, tolerated base pairs (A–C and G–U) between *NbGSA* transcript and NtsiRNA are also shown by orange background.
(TIF)

Figure S2 **Transgenic** ***N. benthamiana*** **by 35S:GS-IR and CoYMV:GS-IR.** (a) 35S:GS-IR, (b) CoYMV:GS-IR, leaf of (c) Empty, (d) 35S:GS-IR, and (e) CoYMV:GS-IR. Bar is 1 cm. Transversal section in the vicinity of a minor leaf vein (arrow head) of (f) Empty and (g) CoYMV:GS-IR, and their chlorophyll fluorescence images of (h) Empty and (i) CoYMV:GS-IR. Bar is 0.1 mm. (j) qRT-PCR analysis of *NbGSA* and *NbSu-s* mRNA in the transgenic plants. The data are shown with SD of three technical replicates. (k) Northern blot analysis of *NtGSA* siRNAs in the transgenic leaves with *NtGSA* antisense probe.
(TIF)

Figure S3 **Chlorophyll amount in the emerged leaves of agroinfiltrated WT.** Samples were taken from three locations per a leaf (L2 of Figure 3b). The relative amounts of five independent plants are shown with SD; the level of an Empty was set at 100. Asterisks indicate significant difference from the Empty (*p<0.5, **p<0.01).
(TIF)

Figure S4 **Northern blot analysis of** ***NbGSA*** **3′ region siRNA in transgenic plants and agroinfiltrated plants.** (a) The location of the *NbGSA* 3′ region probe used. (b) Absence of the hybridizing signals in respective samples.
(TIF)

Table S1 **Sequences of primers used in PCR and qRT-PCR.**
(DOC)

## References

[1] Sjölund RD (1997). The phloem sieve element: a river runs through it.. Plant Cell.

[2] Oparka KJ, Turgeon R (1999). Sieve elements and companion cells - traffic control centers of the phloem.. Plant Cell.

[3] Suárez-López P (2005). Long-range signaling in plant reproductive development.. Int J Dev Biol.

[4] Kehr J, Buhtz A (2008). Long distance transport and movement of RNA through the phloem.. J Exp Bot.

[5] Kragler F (2010). RNA in the phloem: A crisis or a return on investment?. Plant Sci.

[6] Harada T (2010). Grafting and RNA transport via phloem tissue in horticultural plants.. Scientia Horticulturae.

[7] Mudge K, Janick J, Scofield S, Goldschmidt EE, Janick J (2009). A history of grafting.. In Horticultural reviews.

[8] Waterhouse PM, Wang M-B, Lough T (2001). Gene silencing as an adaptive defence against viruses.. Nature.

[9] Dunoyer P, Brosnan CA, Schott G, Wang Y, Jay F (2010). An endogenous, systemic RNAi pathway in plants.. EMBO J.

[10] Molnar A, Melnyk CW, Bassett A, Hardcastle TJ, Dunn R (2010). Small silencing RNAs in plants are mobile and direct epigenetic modification in recipient cells.. Science.

[11] Palauqui JC, Vaucheret H (1998). Transgenes are dispensable for the RNA degradation step of cosuppression.. Proc Natl Acad Sci USA.

[12] Voinnet O, Lederer C, Baulcombe DC (2000). A viral movement protein prevents spread of the gene silencing signal in *Nicotiana benthamiana*.. Cell.

[13] Allison R, Thompson C, Ahlquist P (1990). Regeneration of a functional RNA virus genome by recombination between deletion mutants and requirement for cowpea chlorotic mottle virus 3a and coat genes for systemic infection.. Proc Natl Acad Sci USA.

[14] Höfgen R, Axelsen KB, Kannangara CG, Schüttke I, Pohlenz H-A (1994). A visible marker for antisense mRNA expression in plants: Inhibition of chlorophyll synthesis with a glutamate-1-semialdehyde aminotransferase antisense gene.. Proc Natl Acad Sci U S A.

[15] Matsuda Y, Liang G, Zhu Y, Ma F, Nelson RS (2002). The commelina yellow mottle virus promoter drives companion-cell-specific gene expression in multiple organs of transgenic tobacco.. Protoplasma.

[16] Stadler R, Sauer N (1996). The *Arabidopsis thaliana* AtSUC2 gene is specifically expressed in companion cells.. Bot Acta.

[17] Hedtke B, Alawady A, Chen S, Börnke F, Grimm B (2007). HEMA RNAi silencing reveals a control mechanism of ALA biosynthesis on Mg chelatase and Fe chelatase.. Plant Mol Biol.

[18] Crété P, Leuwnberger S, Iglesian VA, Suarez V, Schöb H (2001). Graft transmission of induced and spontaneous post-transcriptional silencing of chitinase genes.. Plant J.

[19] Tournier B, Tabler M, Kalantidis K (2006). Phloem flow strongly influences the systemic spread of silencing in GFP *Nicotiana benthamiana* plants.. Plant J.

[20] Brosnan CA, Mitter N, Christie M, Smith NA, Waterhouse PM (2007). Nuclear gene silencing directs reception of long-distance mRNA silencing in *Arabidopsis*.. Proc Natl Acad Sci USA.

[21] Himber C, Dunoyer P, Moissiard G, Ritzenthaler C, Voinnet O (2003). Transitivity-dependent and -independent cell-to-cell movement of RNA silencing.. EMBO J.

[22] Xu H, Zhang W, Li M, Harada T, Han Z, Li T (2010). *GIBBERELLIC ACID INSENSITIVE* mRNA transport in both directions between stock and scion in *Malus*.. http://dx.doi.org/10.1007/s11295-010-0309-7.

[23] Kanehira A, Yamada K, Iwaya T, Tsuwamoto R, Kasai A (2010). Apple phloem cells contain some mRNAs transported over long distances.. Tree Genet Genom.

[24] Chitwood DH, Nogueira TS, Howell MD, Montgomery TA, Carrington JC (2009). Pattern formation via small RNA mobility.. Genes Devel.

[25] Carlsbecker A, Lee J-Y, Roberts CJ, Dettmer J, Lehesranta S (2010). Cell signaling by microRNA165/6 directs gene dose-dependent root cell fate.. Nature.

[26] Pant BD, Buhtz A, Kehr J, Scheible WR (2007). MicroRNA399 is a long-distance signal for the regulation of plant phosphate homeostasis.. Plant J.

[27] Lin SI, Chiang SF, Lin WY, Chen JW, Tseng CY (2008). Regulatory network of microRNA399 and PHO2 by systemic signaling.. Plant Physiol.

[28] Martin A, Adam H, Diaz-Mendoza M, Żurczak M, González-Schain D (2009). Graft-transmissible induction of potato tuberization by the microRNA miR172.. Development.

[29] Buhtz1 A, Pieritz J, Springer F, Kehr1 J (2010). Phloem small RNAs, nutrient stress responses, and systemic mobility.. BMC Plant Biology.

[30] Mitsuhara M, Ugaki H, Hirochika M, Ohshima T, Murakami Y (1996). Efficient promoter cassettes for enhanced expression of foreign genes in dicotyledonous and monocotyledonous plants.. Plant Cell Physiol.

[31] Kasai A, Kanehira A, Harada T (2010). *miR172* can move long distances in *Nicotiana benthamiana*.. Open Plant Sci J.

[32] Murashige T, Skoog F (1962). A revised medium for rapid growth and bioassays with tobacco cultures.. Physiol Plant.

[33] Porra RJ, Thomson WA, Kriedemann PE (1989). Determination of accurate extinction coefficients and simultaneous equations for assaying chlorophylls *a* and *b* extracted with four different solvents: verification of the concentration of chlorophyll standards by atomic absorption spectroscopy.. Biochim Biophys Acta.

