# Supplementary figures and images for: Graft-Transmitted siRNA Signal from the Root Induces Visual Manifestation of Endogenous Post-Transcriptional Gene Silencing in the Scion
Source: PLoS One. 2011 Feb 9;6(2):e16895. doi: 10.1371/journal.pone.0016895 (PMC3036722; doi:10.1371/journal.pone.0016895)

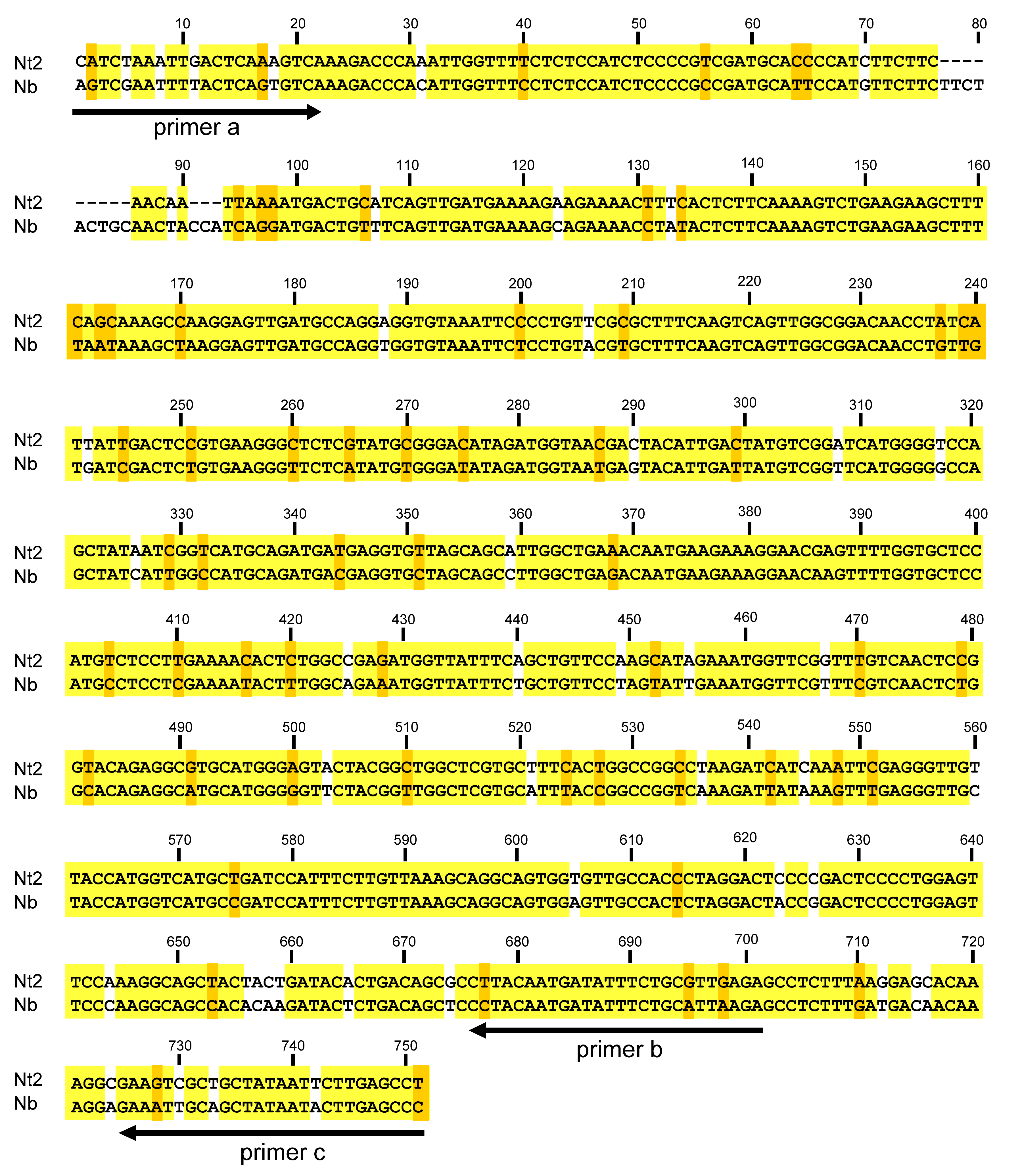

Supplement: Figure S1 — cDNA alignment between N. tabacum GSA2 (Accession No. x65974) and N. benthamiana orthologous GSA. Amplified region of NtGSA by primers a and b was used as the inverted repeat of the GSA; GS-IR. The sequence from 701 to 751 was the loop frame. On mismatched base pairs, tolerated base pairs (A–C and G–U) between NbGSA transcript and NtsiRNA are also shown by orange background. (TIF) [file pone.0016895.s001.tif]

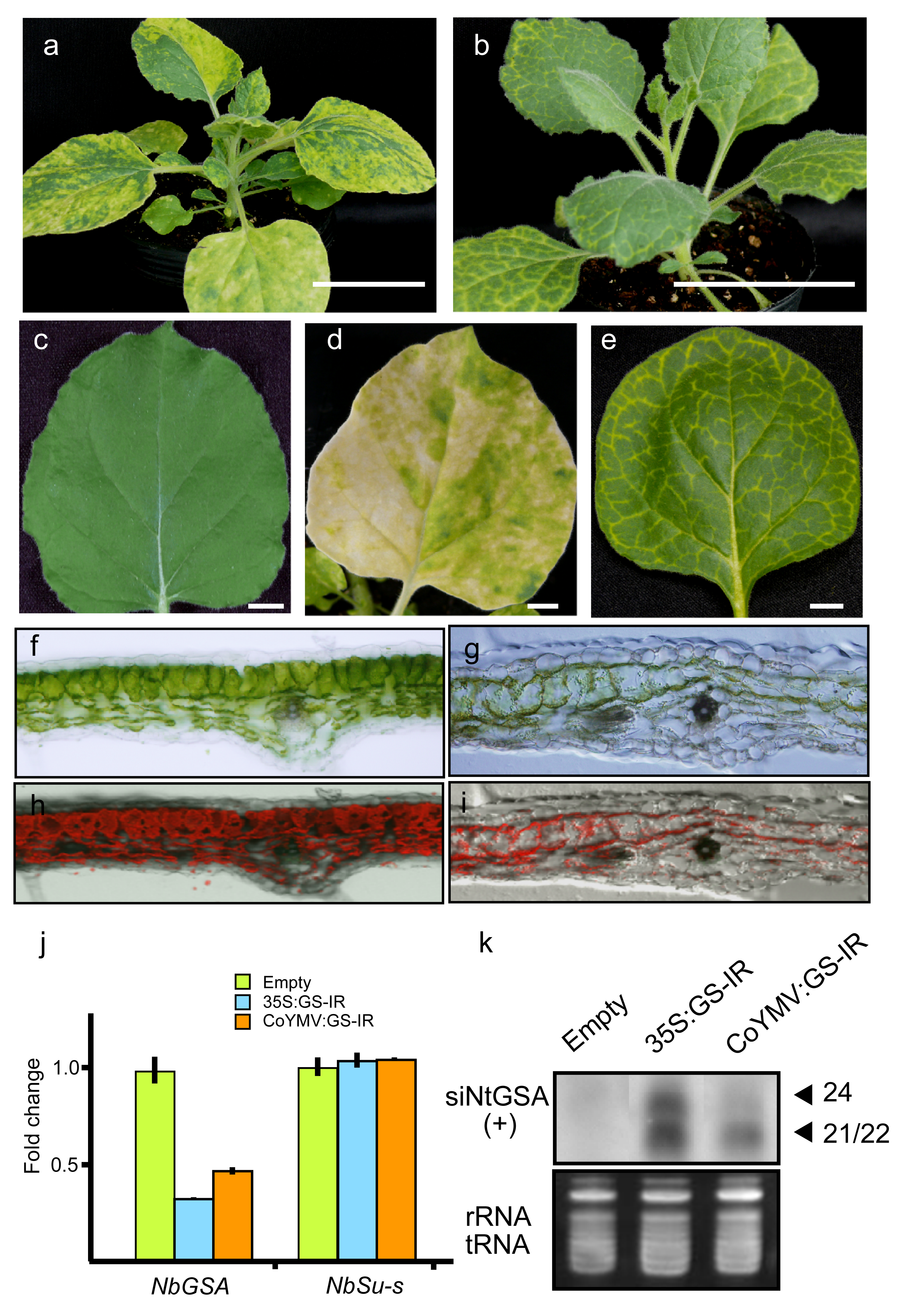

Supplement: Figure S2 — Transgenic N. benthamiana by 35S:GS-IR and CoYMV:GS-IR. (a) 35S:GS-IR, (b) CoYMV:GS-IR, leaf of (c) Empty, (d) 35S:GS-IR, and (e) CoYMV:GS-IR. Bar is 1 cm. Transversal section in the vicinity of a minor leaf vein (arrow head) of (f) Empty and (g) CoYMV:GS-IR, and their chlorophyll fluorescence images of (h) Empty and (i) CoYMV:GS-IR. Bar is 0.1 mm. (j) qRT-PCR analysis of NbGSA and NbSu-s mRNA in the transgenic plants. The data are shown with SD of three technical replicates. (k) Northern blot analysis of NtGSA siRNAs in the transgenic leaves with NtGSA antisense probe. (TIF) [file pone.0016895.s002.tif]

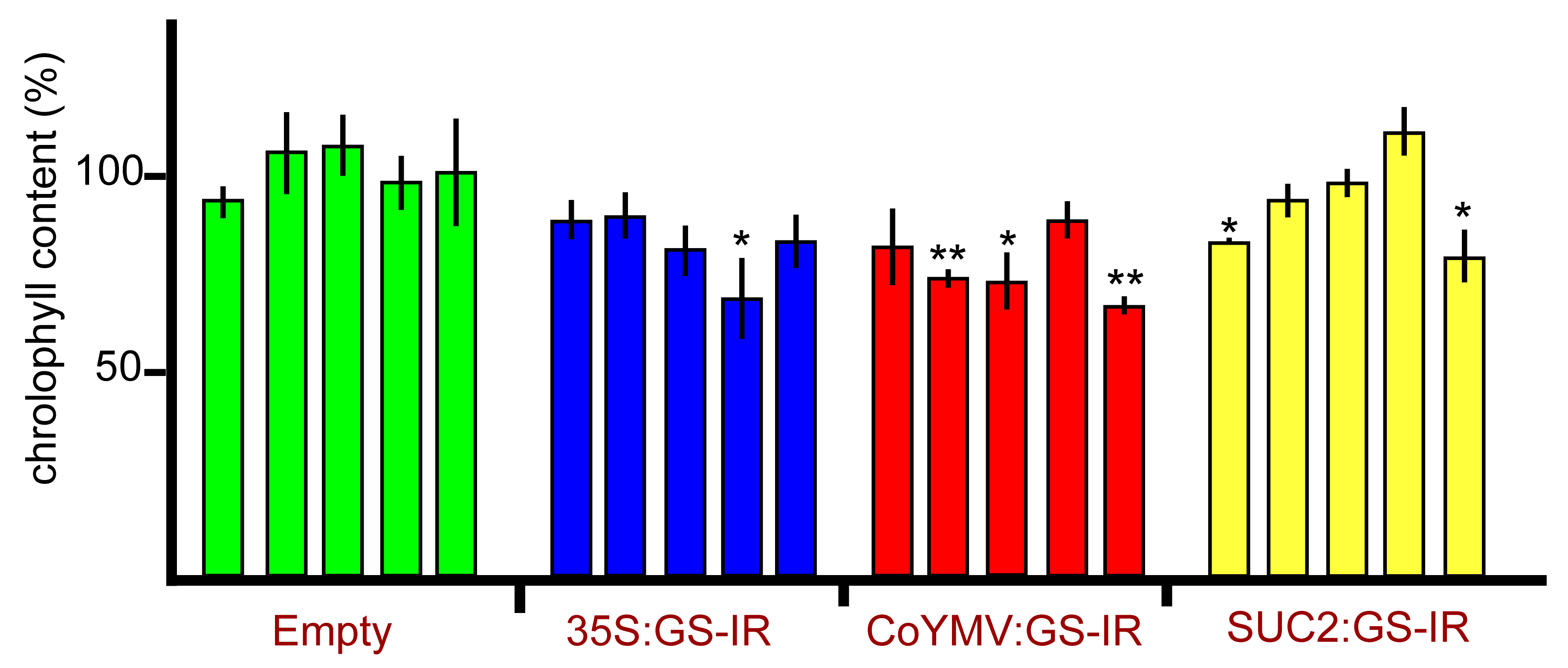

Supplement: Figure S3 — Chlorophyll amount in the emerged leaves of agroinfiltrated WT. Samples were taken from three locations per a leaf (L2 of Figure 3b). The relative amounts of five independent plants are shown with SD; the level of an Empty was set at 100. Asterisks indicate significant difference from the Empty (*p<0.5, **p<0.01). (TIF) [file pone.0016895.s003.tif]

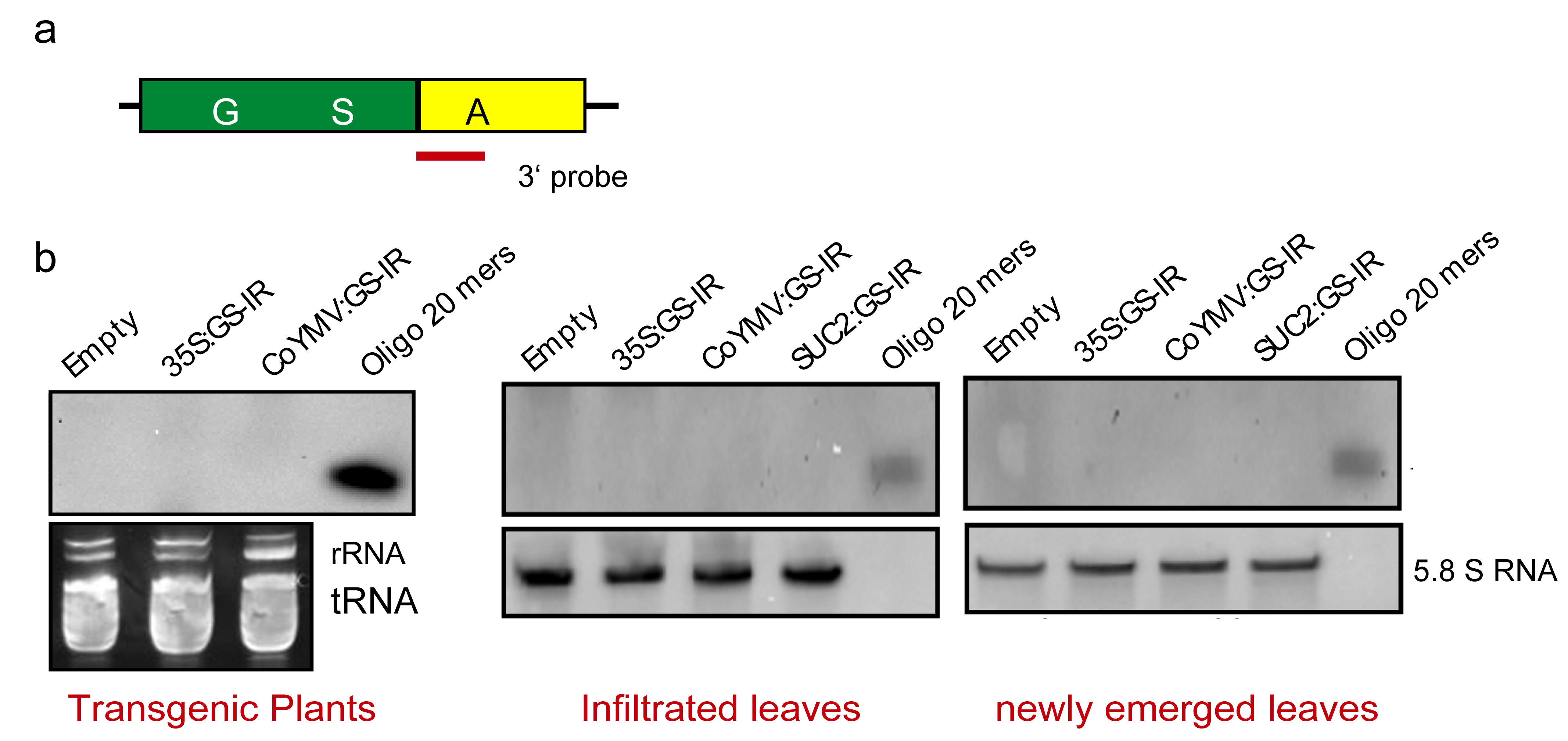

Supplement: Figure S4 — Northern blot analysis of NbGSA 3′ region siRNA in transgenic plants and agroinfiltrated plants. (a) The location of the NbGSA 3′ region probe used. (b) Absence of the hybridizing signals in respective samples. (TIF) [file pone.0016895.s004.tif]
