# Supplementary material for: Graft-Transmitted siRNA Signal from the Root Induces Visual Manifestation of Endogenous Post-Transcriptional Gene Silencing in the Scion
Source: PLoS One. 2011 Feb 9;6(2):e16895. doi: 10.1371/journal.pone.0016895 (PMC3036722; doi:10.1371/journal.pone.0016895)
Supplement: Table S1 — Sequences of primers used in PCR and qRT-PCR. (DOC) [file pone.0016895.s005.doc]

Table S1 Sequences of primers used in PCR and qRT-PCR

| Name | Sequence^a^ |
| --- | --- |
| *Plasmid　construction*  GSA a  GSA b  GSA c  GSA d  PCoYMV F  PCoYMV R  PSUC2 F  PSUC2 R  ***Probe preparation***  NbGSA 3'F  NbGSA 3'R  5.8S F  5.8S R  ***qRT-PCR***  NbGSA F  NbGSA R  NbSu-s F  NbSu-s R  NbUbi F  NbUbi R | 5'-AAGGATCCTCATCTAAATTGACTCAAAGTCA -3'  5'- AATCTAGACTCTTGCGAATGGCAGCAAG -3'  5'- AATCTAGACTAAATCCAGTCATGACTTC -3'  5'- AAGAGCTCTCATGGCCATCTAAATTGACTC -3'  5'- GCGTCGACGGTATCGATTTCTTAGG -3'  5'- GCGGATCCTTGTTGTGTTGGGTTTTC -3'  5'- GCGTCGACATGTAACTACTTTGCTTATGTG -3'  5'- GAGGATCCATTTGACAAACCAAGAAAGTA -3'  5'- GTTGGCATATGGTGGCGCT -3'  5'- CCAAGTGTTCATATGCTCCC -3'  5'- CGTAGCGAAATGCGATACTTGGTG -3'  5'- GGCCAACCGCACGCTCGAGG -3'  5'- TTCAAGTCAGTTGGCGGACAA -3'  5'- GGAGCACCAAAACTTGTTCC -3  5'- ACTTGCTGAGGAGAGCCAGA -3  5'- TGAATTGAACGGATCACCAG -3'  5'- CGAACCCTAGCCGATTACAA -3'  5'- TCCTTACGAAGCCTCTGGAC -3' |

a Extra restriction enzyme site added is undrelined
